# Evidence inconclusive - comment on article by Schoenfeld et al

**DOI:** 10.1186/s12970-016-0148-5

**Published:** 2016-10-10

**Authors:** David J. Beale

**Affiliations:** OptumRx, Home Delivery, Pharmacist, United Health Group, 2300 Main St, Irvine, CA 92614 USA

## Abstract

This article comments on the study by Schoenfeld, et al. entitled “The effect of protein timing on muscle strength and hypertrophy: a meta-analysis” and discusses how the methodology led to inconclusive results.

Recently, Journal of the International Society of Sports Nutrition published a meta-analysis to determine whether consuming protein in and around a workout enhanced strength and muscular development [1]. In the paper, the authors argue that the results refute the claim that protein intake timing around training sessions positively influences strength and muscular adaptations. However, most of the studies chosen for analysis were not even suitable to answer the study question.

Twenty of the 23 studies in the meta-analysis compared protein supplement to placebo and of these 20 studies none matched total daily protein intake between groups. Therefore, the studies afford no reasonable assessment of whether timing of protein intake around exercise is beneficial because higher total daily protein intake is a confounding factor. Only 3 studies in the meta-analysis would be applicable to the question of protein timing [2–4]. This is because the studies compared protein supplement intake in and around workouts to supplement intake some time before and after. The sample sizes were small and the 3 studies comprised a total of 77 subjects. The authors’ assertion that the study had “good statistical power” due to the sample size of about 500 subjects is belied by the use of only 3 relevant studies.
